# Infographic: A Holistic Perspective of the Societal Relevance of Beef Production and Its Impacts On Climate Change

**DOI:** 10.1093/jas/skad109

**Published:** 2023-04-20

## Abstract

**Figure:**
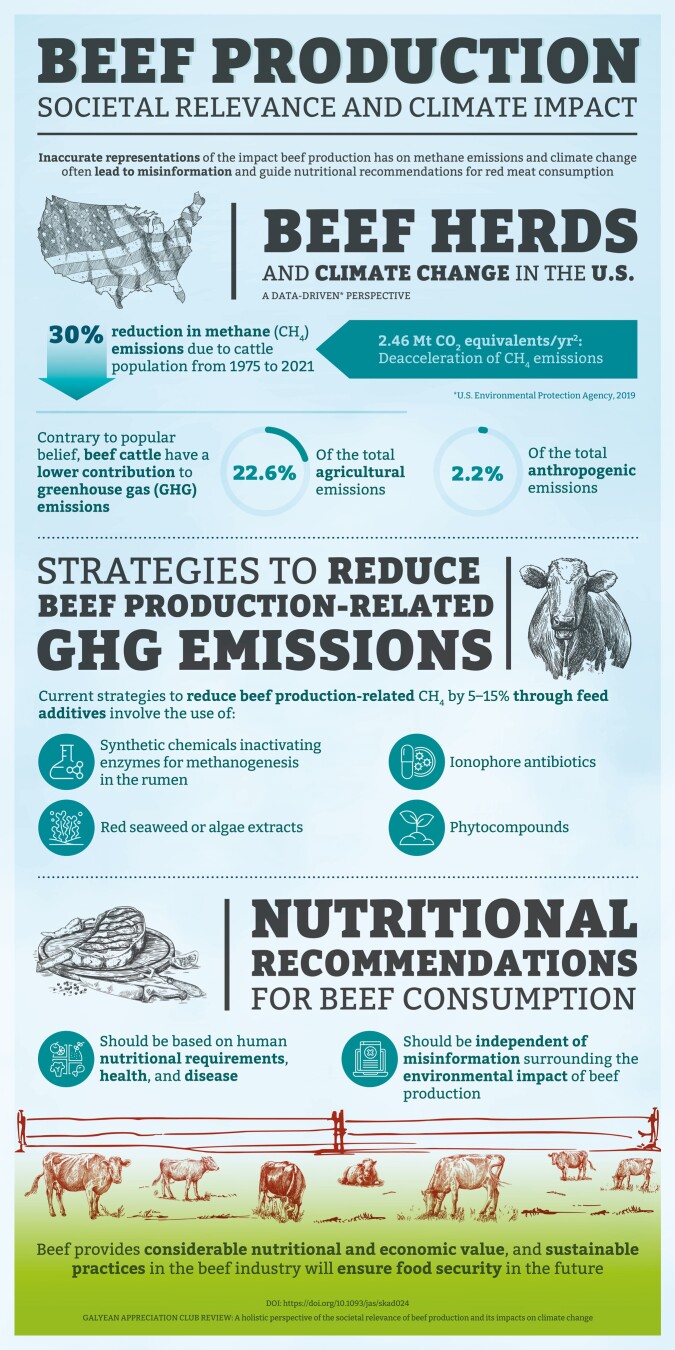

This infographic highlights a recently published paper titled “A Holistic Perspective of the Societal Relevance of Beef Production and Its Impacts On Climate Change” (Tedeschi and Beauchemin, 2023). A key component of the paper is the discussion about the relevance of the U.S. beef cattle industry to society and on its contribution to greenhouse gas emissions. This paper provides some important and provocative insight into the role of beef production, its environmental impacts and current nutritional recommendations for humans.
